# LeadOp+R: Structure-Based Lead Optimization With Synthetic Accessibility

**DOI:** 10.3389/fphar.2018.00096

**Published:** 2018-03-05

**Authors:** Fang-Yu Lin, Emilio Xavier Esposito, Yufeng J. Tseng

**Affiliations:** ^1^Graduate Institute of Biomedical Electronics and Bioinformatics, National Taiwan University, Taipei, Taiwan; ^2^exeResearch, LLC, East Lansing, MI, United States; ^3^Department of Computer Science and Information Engineering, National Taiwan University, Taipei, Taiwan

**Keywords:** fragment-based, lead optimization, structure-based drug design, computer-assisted synthesis, human 5-lipoxygenase, tie-2 kinase

## Abstract

We previously described a structure-based fragment hopping for lead optimization using a pre-docked fragment database, “LeadOp,” that conceptually replaced “bad” fragments of a ligand with “good” fragments while leaving the core of the ligand intact thus improving the compound's activity. LeadOp was proven to optimize the query molecules and systematically developed improved analogs for each of our example systems. However, even with the fragment-based design from common building blocks, it is still a challenge for synthesis. In this work, “LeadOp+R” was developed based on 198 classical chemical reactions to consider the synthetic accessibility while optimizing leads. LeadOp+R first allows user to identify a preserved space defined by the volume occupied by a fragment of the query molecule to be preserved. Then LeadOp+R searches for building blocks with the same preserved space as initial reactants and grows molecules toward the preferred receptor-ligand interactions according to reaction rules from reaction database in LeadOp+R. Multiple conformers of each intermediate product were considered and evaluated at each step. The conformer with the best group efficiency score would be selected as the initial conformer of the next building block until the program finished optimization for all selected receptor-ligand interactions. The LeadOp+R method was tested with two biomolecular systems: Tie-2 kinase and human 5-lipoxygenase. The LeadOp+R methodology was able to optimize the query molecules and systematically developed improved analogs for each of our example systems. The suggested synthetic routes for compounds proposed by LeadOp+R were the same as the published synthetic routes devised by the synthetic/organic chemists.

## Introduction

We recently reported a new structure-based fragment hopping method in lead optimization, LeadOp, (Lin and Tseng, 2011). Our lead optimization method works by decomposing a chemical structure into fragments of different parts, either by chemical or user-defined rules. The fragments are evaluated in a pre-docked fragment database and ranked according to specific fragment-receptor binding interactions. The ranked fragments provide the ability to replace fragments possessing less favorable contributions to binding. With optimal fragments selected, LeadOp reassembles the fragments to form the new drug-like compound. LeadOp is an algorithm that can automatically optimize a query molecule by searching and replacing fragments from a pre-docked fragment database in the active site to generate structures with better binding without prior knowledge of better fragments. Additionally, users can specify parts of structures to be optimized based on known interactions or the user's preference. However, the proposed compounds are not always easy to synthesize. In this study, we demonstrate lead optimization with synthetic accessibility, LeadOp+R, an advanced approach for lead optimization with synthetic accessibility.

A basic difficulty in most applications of computer-aided drug design is that designed (suggested) molecules are often of uncertain synthetic accessibility, leading to a slow feedback-improvement loop between the experimental syntheses and modeling design (Hopkins et al., 2004). Various synthetic planning software, WODCA (Ihlenfeldt and Gasteiger, 1996), SYNGEN (Hendrickson and Toczko, 1988), and ROBIA, 4 (Socorro and Goodman, 2006) were developed to provide the synthetic route generation, that involves either searching a database of chemical reactions or transformation rules for reaction centers that match the target compound to propose analogous transformations. Tools in route generation, mostly retrosynthetic software, can suggest routes based on encoded generalized reaction rules to identify those bond disconnections most apt to lead to synthetically accessible precursor structures (Corey et al., 1975; Corey and Jorgensen, 1976) while Hendrickson's group (Hendrickson et al., 1985) developed a logic-based synthesis design method with formalized reaction constraints. A good example of route generation is Route Designer (Law et al., 2009), that use rules describing retrosynthetic transformations automatically generated from reaction database and generates complete synthetic routes for target molecules starting from available reactants. Applications combining the synthetic route designing and de-novo design for the target binding sites have also been developed, such as SPROUT (Mata et al., 1995), which starts from generation of a skeleton followed by atom substitution to convert the solution skeletons to molecules and rank the output from SPROUT according to ease of synthesis. However, the molecules are generated from the ease of synthesis, the desired core of potential inhibitors could not be easily preserved.

To make the synthetic-modeling feedback loop more straightforward, we develop and implement “LeadOp+R”—**Lead Op**timization with synthetic accessibility based on chemical **R**eaction route. LeadOp+R is an algorithm that performs structure-based lead optimization while considering the synthetic reactions from reactants to products according to reaction rules. It takes into consideration the chemical reaction environment; this information is based on known chemical synthesis. The synthetic routes suggested by LeadOp+R are examined to ensure the validity of transformation from one starting reactant into the final product through the use of the LeadOp+R reaction database. The extracted reaction rules in LeadOp+R reaction database do not take into account temporarily or unwanted chemical reactions; on the contrary, these extracted reaction rules consider direct chemical reactions that transform the starting reactants into products. LeadOp+R's algorithm consists of the following five steps: (i) identify a preserved space (defined by the volume occupied by a fragment of the query molecule to be preserved by the user) and searches for building blocks with the same preserved space as initial reactants, (ii) search the reaction rules for each reactants identified, (iii) generate reaction products based on reaction rules, (iv) evaluate the conformations of each products of each reaction, and (v) select the conformer from previous steps that would be selected as the reactant to grow molecules until optimizations are fulfilled for each selected inhibitor–receptor interactions by users. Multiple conformers of each product for each step were considered and evaluated. The conformer with the best group efficiency score would be selected as the next reactant, wherein the group efficiency score is calculated based on binding energy divided by the number of heavy atoms. Thus, this evaluation would favor the conformers with stronger binding toward the specified receptor-ligand interactions with less heavy atoms (Hopkins et al., 2004; Ciulli et al., 2006; Alex and Flocco, 2007; Saxty et al., 2007; Congreve et al., 2008; Orita et al., 2009). Compounds passing the molecular property filters comprised the final list of proposed compounds. The compounds were then energy-minimized and ranked on the basis of the overall ligand–receptor binding energy. To investigate the interactions between the newly assembled molecules and their receptor, molecular dynamics simulations were performed to explore the compounds' poses and interactions with the solved crystal structure of the receptor.

To demonstrate the LeadOp+R algorithm, we selected the Tie-2 kinase (Hodous et al., 2007) and human 5-lipoxygenase (5-LOX) (Ducharme et al., 2010) protein systems and their associated inhibitors as model systems. The endothelium-specific receptor tyrosine kinase Tie-2 (tyrosine kinase containing Ig and EGF homology domains) is primarily expressed in the vascular endothelium and is involved in vessel branching, sprouting, remodeling, maturation, and stability (Yu, 2005). The role of tyrosine kinases in angiogenesis and in the vascularization of solid tumors has drawn considerable interest (Hasegawa et al., 2007) and is considered to be angiogenesis-dependent in cancer. Interference with the Tie-2 pathway by diverse blocking agents has been shown to suppress tumor growth in xenograft studies (Oliner et al., 2004). The development of Tie-2 kinase inhibitors may block the beneficial anti-inflammatory and vascular stabilizing effects, thus the discovery of potent Tie-2 kinase inhibitors has advanced into clinical studies (Huang et al., 2010). Lipoxygenases are a family of iron-containing enzymes found in a large variety of organisms, including bacteria and animals. It catalyzes the dioxygenation of polyunsaturated fatty acids containing a *cis*-1,4-pentadiene structure—the first committed structure in a metabolic pathway cascade—and involved in the initiation of signaling molecule synthesis and inducing structural or metabolic changes (Steele et al., 1999). Four major isozymes of lipoxygenases have been identified (Ivanov et al., 2010), including 5-, 8-, 12-, and 15-LOX, that are key enzymes in the metabolism of prostaglandins and leukotrienes. In particular, leukotrienes are produced through the 5-LOX pathway and the increased activity of the 5-LOX pathway is strongly associated with atherosclerosis (Woods et al., 1993). As the 5-LOX biological pathways and byproducts lead to inflammation, discovering a 5-lipoxygenase inhibitor is important to the fields of inflammatory and allergic diseases (Shaffer and Mansmann, 1997).

## Materials and methods

### Overall procedure

**Figure 1:**
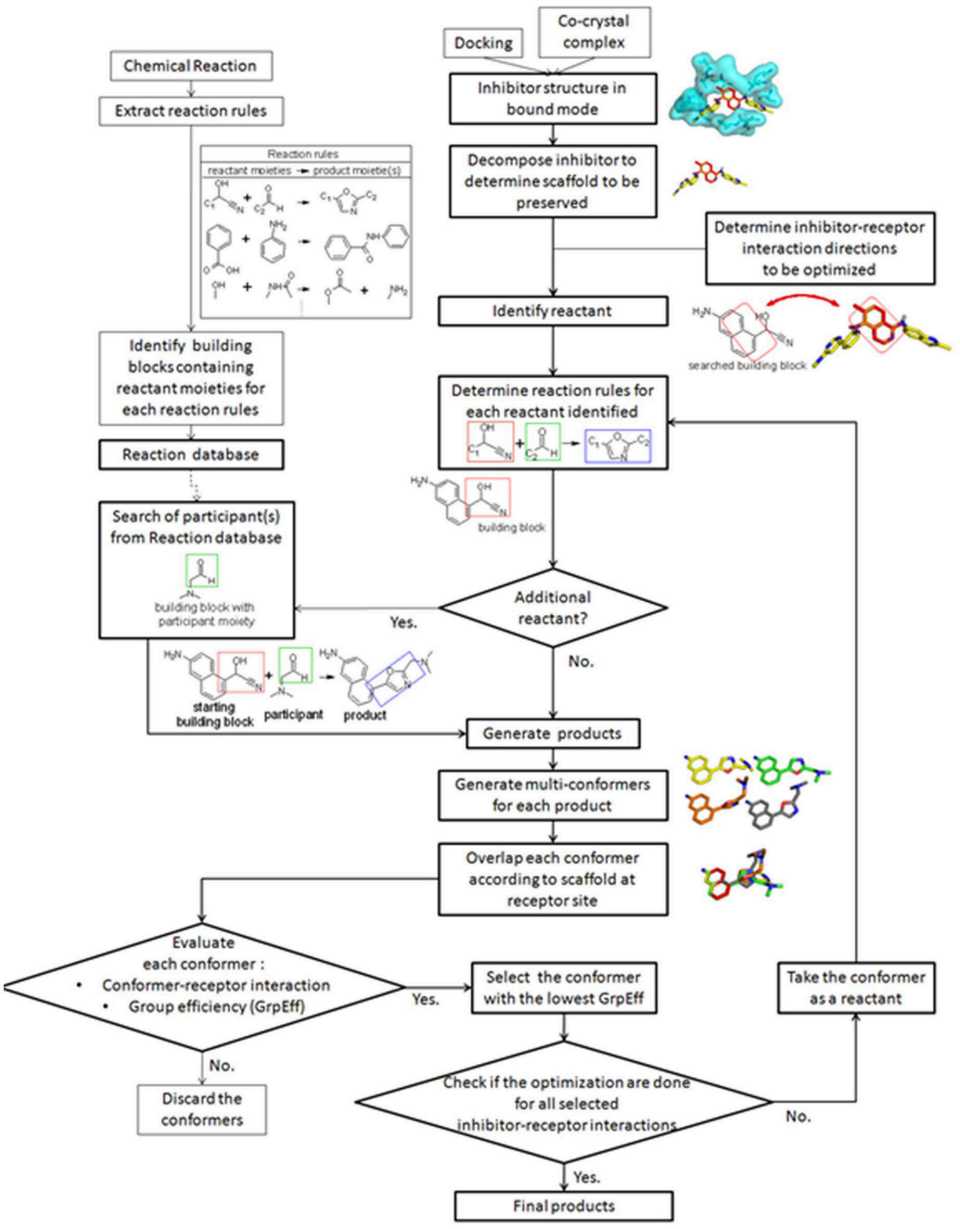
Illustration of the LeadOp+R optimization workflow.

The general protocol for LeadOp+R is illustrated in Figure 1 and the details of each step are described in the following sections. Prior to applying the LeadOp+R optimization procedure, a reaction rule database is constructed, containing reaction rules for the reactant moiety, the product moiety, and the building blocks of each reaction. Thus, participants involved in each reaction are known for synthetic assessment in LeadOp+R. The initial step of LeadOp+R requires the user to select the favored inhibitor-receptor interaction positions for optimization. The inhibitor-receptor interaction positions determine the “direction” for virtual synthesis and optimizations. LeadOp+R will systematically optimize and grow a structure until all the user-defined directions are processed. LeadOp+R initiates the analysis with the inhibitor-receptor complex from docking studies or crystal structures. The user can determine which fragment(s) in the query inhibitor (initial compound) to preserve during optimization. To ensure that the initial synthesis is accessible, the starting building block—containing the preserved fragment—is used as the initial building block. LeadOp+R then searches the reaction rule database with this building block to identify associated reactions rules. Once the reactions rules and associated participants are identified, the products of each reaction rule are generated virtually. The best binding conformation of the proposed compound is selected from an ensemble of conformers are constructed of each compound. The conformer of each compound with the lowest group efficiency value is selected as the initial conformer of the next building block until the program reaches the termination condition. By evaluating the contribution of each product upon binding with group efficiency, LeadOp+R selects compounds that bind stronger yet possess less heavy atoms. The compounds passing a set of molecular property filters comprised the final list of proposed compounds. Following a short molecular dynamics simulation, the compounds are energy-minimized and ranked on the basis of the overall ligand–receptor binding (interaction) energy. This provides a series of new and more potent compounds that are chemical accessibility.

### Example systems

Tie-2 kinase (PDB: 2p4i), an endothelium-specific receptor tyrosine kinase (Hodous et al., 2007) and human 5-LOX enzyme (Charlier et al., 2006) a key enzyme in leukotriene biosynthesis, were selected as model systems to examine the LeadOp+R approach. One Tie-2 kinase inhibitor, compound 46 in reference 16 (denoted as compound rA in this study) and a human 5-LOX inhibitor, compound 7 (substituted coumarins) in reference 17 (denoted as compound rB in this study), were selected as the LeadOp+R optimization examples.

### Construction of the LeadOp+R reaction database

**Figure 2:**
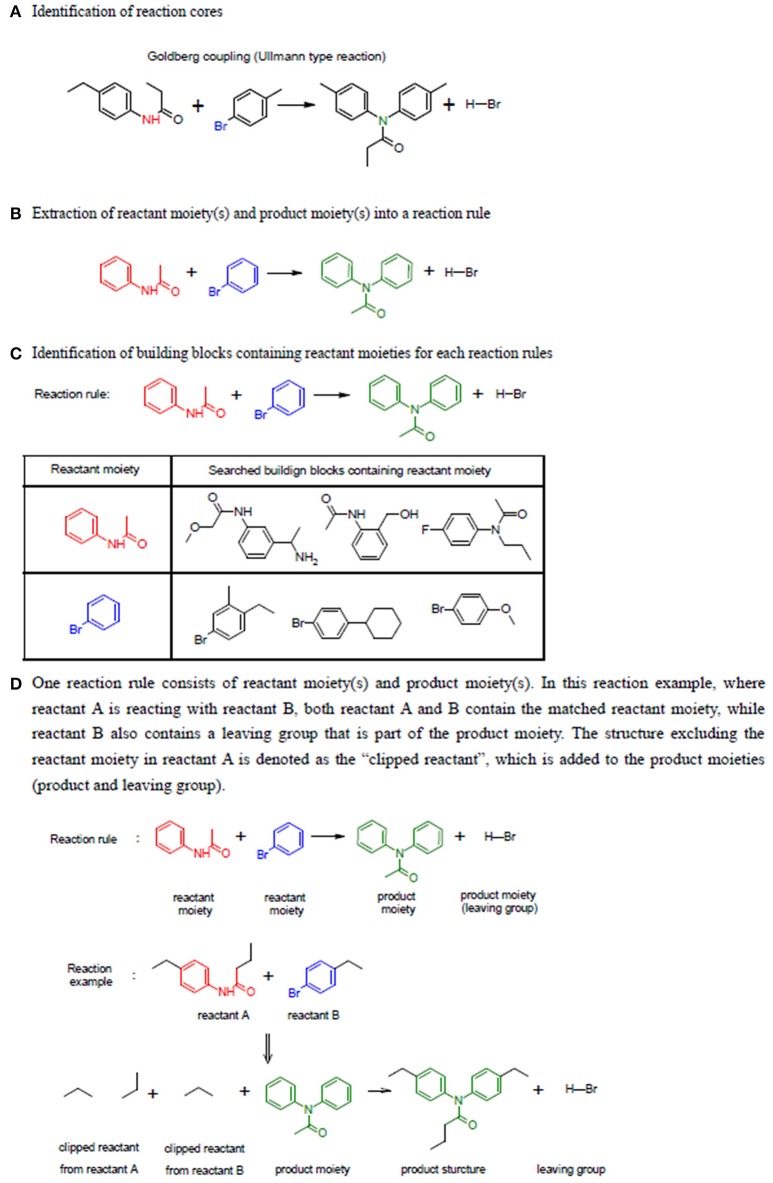
Example of three steps used to construct the table of reaction rules. **(A)** Identification of reaction cores. The atoms with changed atom attributes are highlighted in red and blue within the two reactants. **(B)** Extraction of the moieties. **(C)** Identification of building blocks containing the reactant moieties. **(D)** Illustration of the steps in generating products.

LeadOp+R collects chemical reactions, building blocks, and reaction rules with reactant moieties and product moieties of each reaction to construct the LeadOp+R reaction database. LeadOp+R includes 198 classic chemical reactions from the Reaxy Database and 2,091 organic building blocks from the commercially available Sigma-Alderich Co^1^. product library. These building blocks include the typical building blocks in a chemical synthesis such as various nitrogen compounds (amines, isocyanides) and carbonyl compounds (amides, aldehydes, and ketones). A reaction rule in LeadOp+R includes the reactant moieties and product moieties extracted from the full structure of reactants and products of each reaction collected. In LeadOp+R, the reaction moieties were defined and extracted from a chemical reaction according the following steps (see Figure 2 for the illustration of the steps):

1. Identification of reaction core. A collection of atoms that take part in the chemical transformation (reaction) have their atom type changed (element, number and type of bonds, and number of neighboring atoms) are considered the reaction core. These atoms are determined by comparing the atoms of the starting compound and product to those within the LeadOp+R reaction database; atoms that differ are part of the reaction core. Since the reaction core does not contain enough chemical information to accurately describe the reaction, additional information is gathered from atoms bound to the reaction core.
2. Extraction of the reactant and product moieties for a reaction. The initial reaction cores typically do not include enough atoms and thus their “chemical environment” is expanded. The reaction core is increased to bonded (neighboring) atoms until the minimum reactant and product substructures are included to fully represent the reaction. Within a reaction, the reactant portion is denoted as the “reactant moiety” and as expected the product portion is denoted as “product moiety.” The extension step is performed by traversing the atom types within the reaction core, as discussed in Step 1, until a single sp^3^ carbon is found and the atoms searched during the extension step are considered part of the same moiety. For cases where the searched atoms are in an aromatic ring, the extension was terminated when all the atoms in the aromatic ring are included in the moiety, thus all the atoms in the aromatic ring are considered part of the moiety.

Finally, the building blocks with the same reactant moiety for each reaction rule are collected (through a JChem application-programming interface; JChem API) and classified by the reaction. Building blocks for each reaction rule are recorded and used for virtual synthesis in the LeadOp+R algorithm.

### Identify reactant

LeadOp+R initiates the analysis of a complexed structure (inhibitor-receptor) taken from a docking study or crystal structure. Initially, the user identifies and preserves the “fragment space” of a query molecule defined by a fragment's volume. LeadOp+R then searches for building blocks with the same volume as the potential initial reactants. Products of each potential initial reactant are virtually synthesized according to the steps below. For each product molecule that passes the evaluation step, that product molecule becomes the next reactant in the next synthesis step.

### Determine reaction rules for each reactant identified

When a reactant is identified in the previous step, there are many potential reactant moieties and reactions associated with this reactant. Each reactant is subjected to sub-structure searching^2^ to identify atom arrangements (moieties) that are part of a chemical reaction rule within the LeadOP+R reaction database. This is done to determine potential chemical reactions for this specific reactant.

### Generation of reaction products based on reaction rules

Once all the potential reaction rules of a reactant are identified, the corresponding products are generated by “reacting” the reactant moieties and participant reactants (Figure 2D). In LeadOp+R, each reactant has two parts: one structure matches the reactant moiety and the other structure—excluding the reactant moiety—is denoted as the “clipped reactant.” The same definition is used for other building blocks (participants) involved in a reaction. Each product is generated by combining the clipped portion of the reactant and the clipped portion of the participants as well as the product moiety based on the search of the reaction rule.

### Evaluation of the products for each reaction

**Figure 3:**
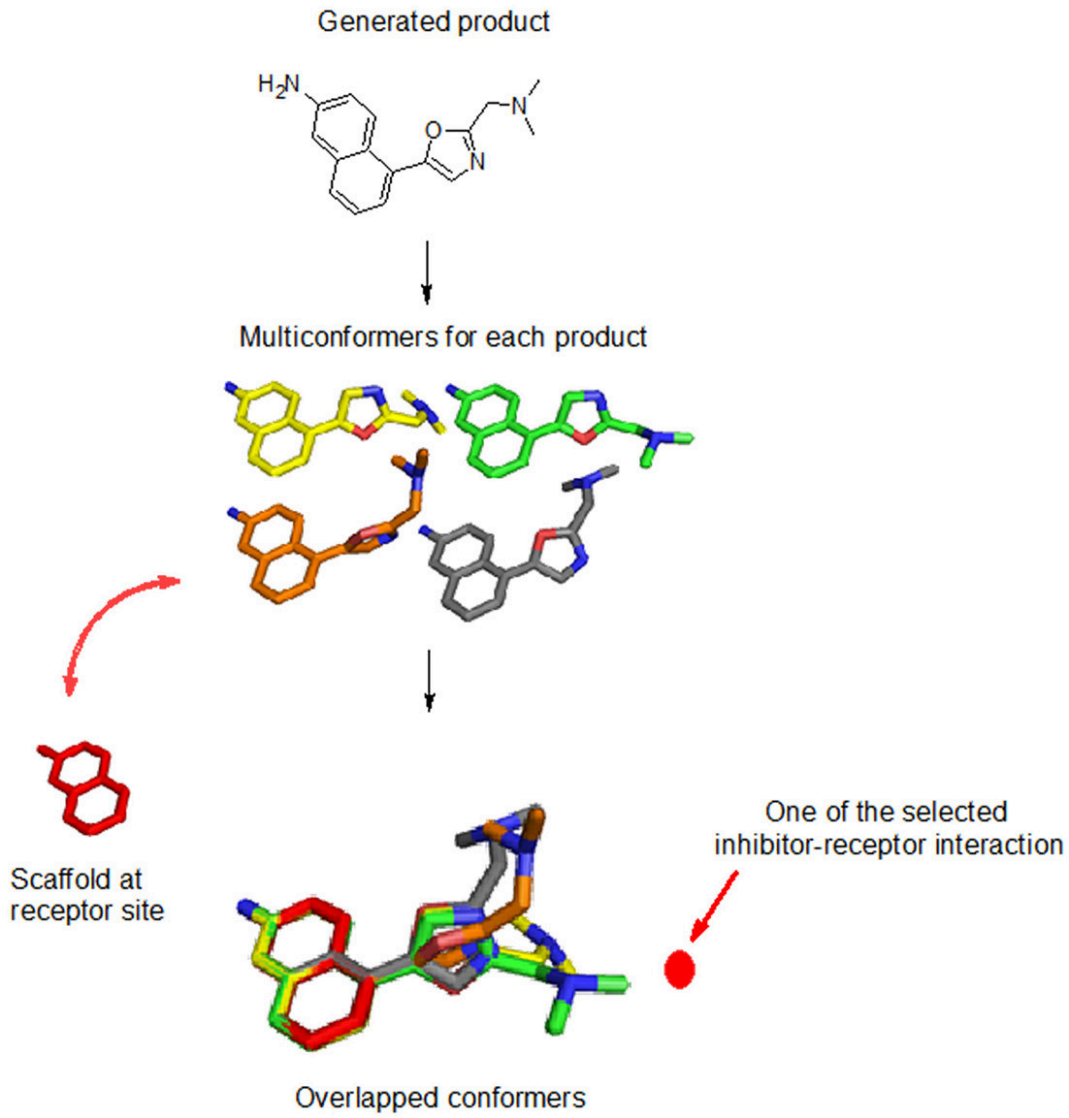
Evaluation of each product for each reaction. Thirty conformers are generated (colored in yellow, green, orange, and gray sticks) and overlaid with the reactant within the binding site (colored in red stick). The user-defined inhibitor-receptor interaction direction (location) is indicated by the dotted red line.

Thirty conformers of each product are generated using the Java and JChem application-programming interface (Imre et al., 2006). Each conformer is aligned with the preserved space of the query molecule, while maximizing the overlap volumes, using the flexible 3D alignment tool of Marvin^3^ (see Figure 3). A conformer for each product was selected for the next step if the following criteria are met: (1) the binding mode of each conformer, aligned with the query molecule within the receptor site, has the same inhibitor-receptor interaction direction, and (2) the new moiety has a group efficiency value <−0.1.

### Final selection by structure-based analysis

The selected conformer for each product is the reactants for the next reaction in the selected inhibitor-receptor interaction direction. The molecule continues to grow until all the inhibitor-receptor interaction directions are exhausted. The collection of potential new compounds is reduced using the following criteria: molecular weight <600 g mol^−1^ and a calculated lipophilicity (cLogP) <5, which is taken into account based on the Lipinski's Rule-of-Five (Lipinski et al., 2001). The compounds that pass the molecular property filters comprised the final list of proposed compounds. These compounds are then energy-minimized within the binding site and ranked based on the overall ligand-receptor binding energy.

### Molecular dynamics simulations

The bound pose of the newly “constructed” compound, as determined with AutoDock Vina (Trott and Olson, 2010), is refined from the lowest binding free energy and the largest number of favorable ligand-receptor interactions within the binding site. The unfavorable contacts between the docked pose of the energy minimized “constructed” compound (fragments connected to the initial core of the compound) and the residues within the binding site are alleviated using molecular dynamics simulations; allowing the ligand-receptor complex to explore the local energy landscape. The best complex pose (ligand-receptor interaction) was selected and molecular dynamics was performed using GROMACS version 4.03 (Hess et al., 2008) and the GROMOS 53A6 force field (Oostenbrink et al., 2005). The complexes are placed in a simple cubic periodic box of SPC216 type water molecules (Berendsen et al., 1981), and the distance between the protein and each edge of the box was set to 0.9 nm. To maintain overall electrostatic neutrality and isotonic conditions, Na^+^ and Cl^−^ ions were randomly positioned within the solvation box. To maintain the proper structure and remove unfavorable van der Waals contacts, a 1,000-step steepest descent energy minimization was employed and terminated when the convergence criteria of an energy difference between subsequent steps differ <1,000 kJ mol^−1^ nm^−1^. Following the energy minimization, the system is subjected to a 1,200 ps molecular dynamics simulation at constant temperature (300 K), pressure (1 atm), and a time step of 0.002 ps (2 fs) with the coordinates of the system recorded every ps.

## Result and discussion

### LeadOp+R optimization for Tie-2 kinase inhibitors

#### Structure-based lead optimization with synthetic routes

**Figure 4:**
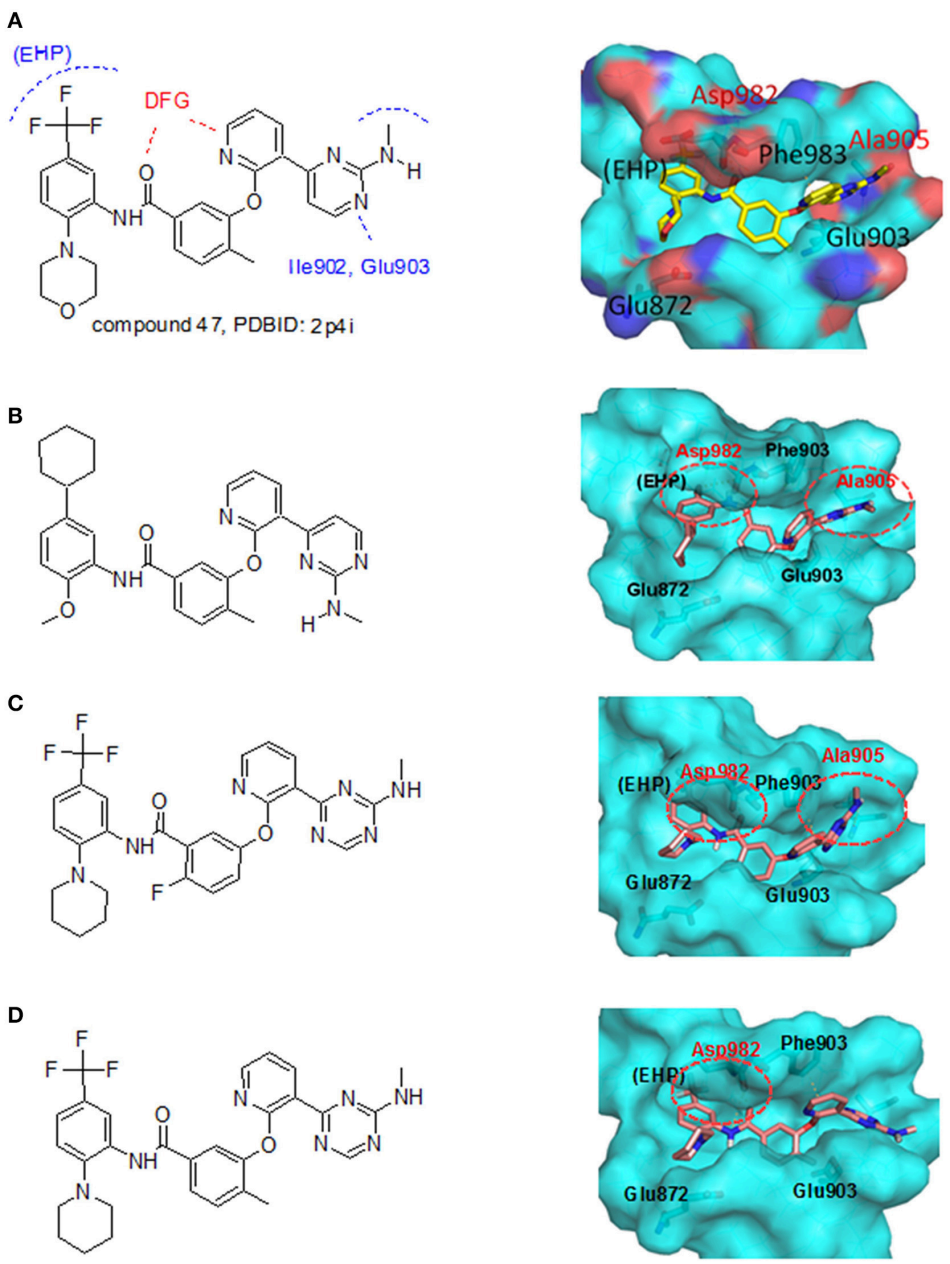
LeadOp+R result for the Tie-2 model system. **(A)** Chemical characteristic of each residue and interaction within the complex of compound 47 from the co-crystal structure (PDB code: 2p4i). **(B–D)** Chemical structure (left) and MDS result (right) of the generated compound rA1 **(B)**, the generated compound rA2 **(C)**, and the generated compound rA3 **(D)**. Carbon atoms are colored pink. Amino acid residues that participate in hydrogen-bonding interactions (labeled red) with the proposed compound at the binding site are depicted with cyan molecular surface.

From the literature (Bridges, 2001), it is known that a good kinase inhibitors should possess a hydrogen-bond donor/acceptor/donor motif to best interact with the backbone carbonyl/NH(amide)/carbonyl presented in the ATP-binding cleft. In the case of Tie-2 kinase, the residues in the active site of the ATP-binding cleft are Ala905 (carbonyl and amide NH) and Glu903 (carbonyl). Additionally, two hydrophobic pockets are part of the active site in the Tie-2 receptor and are designated as the first hydrophobic pocket (HP) and the extended hydrophobic pocket (EHP). We selected a series of Tie-2 inhibitors from the literature (Hodous et al., 2007) containing a co-crystal structure of inhibitor compound 47 with Tie-2 receptor (PDB code: 2p4i). In this co-crystal structure, the 2-(methylamino)pyrimidine ring of inhibitor compound 47 interacts with residue Ala905 via two hydrogen bonds and the pyrimidine is also within van der Waals contact of the Glu903. The central methyl-substituted aryl ring of compound 47 resides in the first hydrophobic pocket (HP), while the pyridine ring forms an edge-to-face π-stacking interaction with Phe983 of the DFG-motif. The carbonyl oxygen makes a hydrogen bond with the backbone NH of Asp982 (DFG motif) and the aryl amide moiety directs the terminal CF_3_-substituted aromatic ring into the EHP. Figure 4A illustrates the ligand-protein interaction of this co-crystal structure.

To demonstrate how LeadOp+R optimizes a compound automatically while considering the potential synthetic route, compound 46 is the query molecule for lead optimization (denoted as compound rA in this study) with a biologically determined IC_50_ value of 399 nM (Hodous et al., 2007). Compound rA was docked into the Tie-2 binding site and the lowest energy conformation was selected. The selected conformation possessed similar molecular interactions, as discussed earlier, with the Tie-2 active site (Figure 4A). The amide functional group of compound rA forms a hydrogen bond with the backbone amide of Asp982, while the pyridine and benzene rings extend into the hydrophobic pocket (HP) and EHP, respectively. The aminobenzoic fragment was designated as the preserved space in this example of LeadOp+R due to the important hydrogen bonding.

To evaluate our algorithm, we compared all of the LeadOp+R generated compounds to Tie-2 kinase inhibitor from the literature and found nine of the LeadOp+R compounds have also been synthesized and their ability to inhibit Tie-2 kinase measured. The inclusive synthesis of proposed products in each LeadOp+R step combined with systematically examining the proposed ligand-receptor interactions resulted in nine compounds with more potent IC_50_ values than the original compound (compound rA). All the LeadOp+R generated compounds were energy minimized in the active site of Tie-2, and then ranked on the basis of the overall ligand–receptor interaction energy. Among all LeadOp+R suggested compounds, nine compounds were previously studied in the literature (Hodous et al., 2007), and the priority suggested by the calculated binding energy had same trend as the experimentally determined IC_50_ values. In this study of Tie-2 kinase inhibitor design—three compounds, denoted as compounds rA1, rA2, and rA3 of the nine LeadOp+R generated compounds—were selected for further investigation. For these three compounds we found detailed synthetic route information (Hodous et al., 2007) and inhibition potency in the literature. These three compound rA1-rA3, have a higher potency than the query compound rA and the suggested priority of the new compounds with the calculated binding energy have a similar IC_50_ potency trend. Depicted representations of compounds rA1-rA3, as well as the corresponding inhibition data from the biological experiments and their predicted binding energy are provided in Table 1.

**Table 1:** Rank of the proposed LeadOp+R compounds based on the calculated binding energy, inhibition concentration (IC_50_) of Tie-2 from the literature (Hodous et al., 2007).

**Rank**	**Structure**	**Inhibition IC_50_ (nM)**
Query	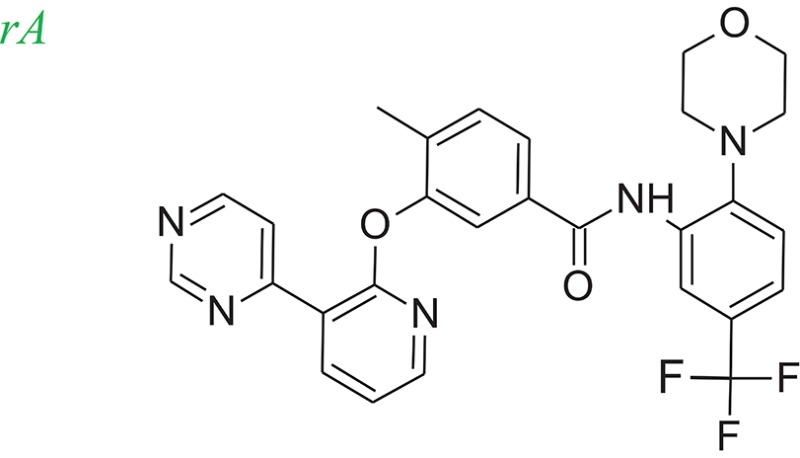	399
38	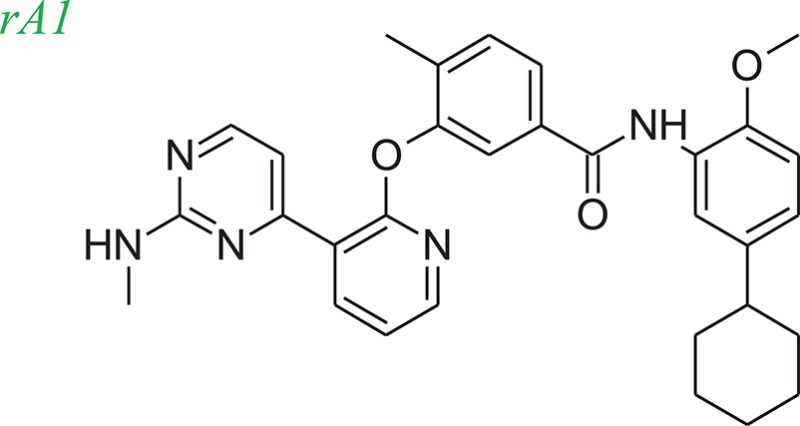	4
113	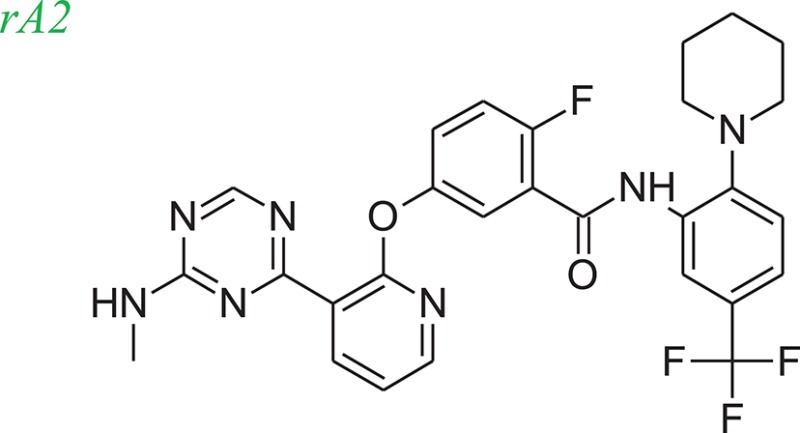	30
292	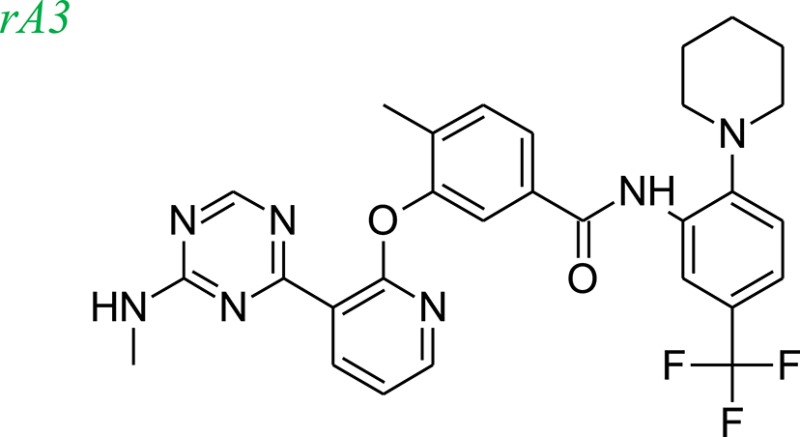	108

*All proposed compounds have a lower IC_50_ value than the query compound and the suggested priority of the three new compounds (out of 631) have a similar trend as the IC_50_ potency values*.

Molecular dynamics simulations were performed with three LeadOp+R generated compounds, rA1–rA3, to further analyze the ligand-protein interactions within the Tie-2 kinase active site. Following geometry optimization of the compounds with respect to Tie-2, molecular dynamics simulation studies were performed and the unique low-energy conformations of the complexes, from the final 50 ps of the MDS (50 configurations), are shown in Figures 4B–D.

In the generated compounds (rA1, rA2, and rA3) both amide arrangements are engaged in strong hydrogen bonds with Asp982 of the DFG-motif (first three residues of the activation loop). The pyrimidine ring in compounds rA1 and rA2 makes key hydrogen bonds with the backbone amide of the linker residue Ala905, situating the pyridine rings in alignment and within edge-to-face π-stacking distance of Phe983 of the DFG-motif. Additionally, the central and terminal aryl rings overlap with only slight differences in orientation for compounds rA1, rA2, and rA3. The additional a hydrogen bond forms between the methoxy group of compound rA1 and residue Asp982, while the CF_3_-groups are placed in essentially the same location within the EHP for compounds rA2 and rA3. These optimized results indicate the hydrogen-bonding and hydrophobic interactions are important for ligands binding to and inhibiting Tie-2, as previously reported (Hodous et al., 2007).

#### Synthetic routes suggested by LeadOp+R

**Figure 5:**
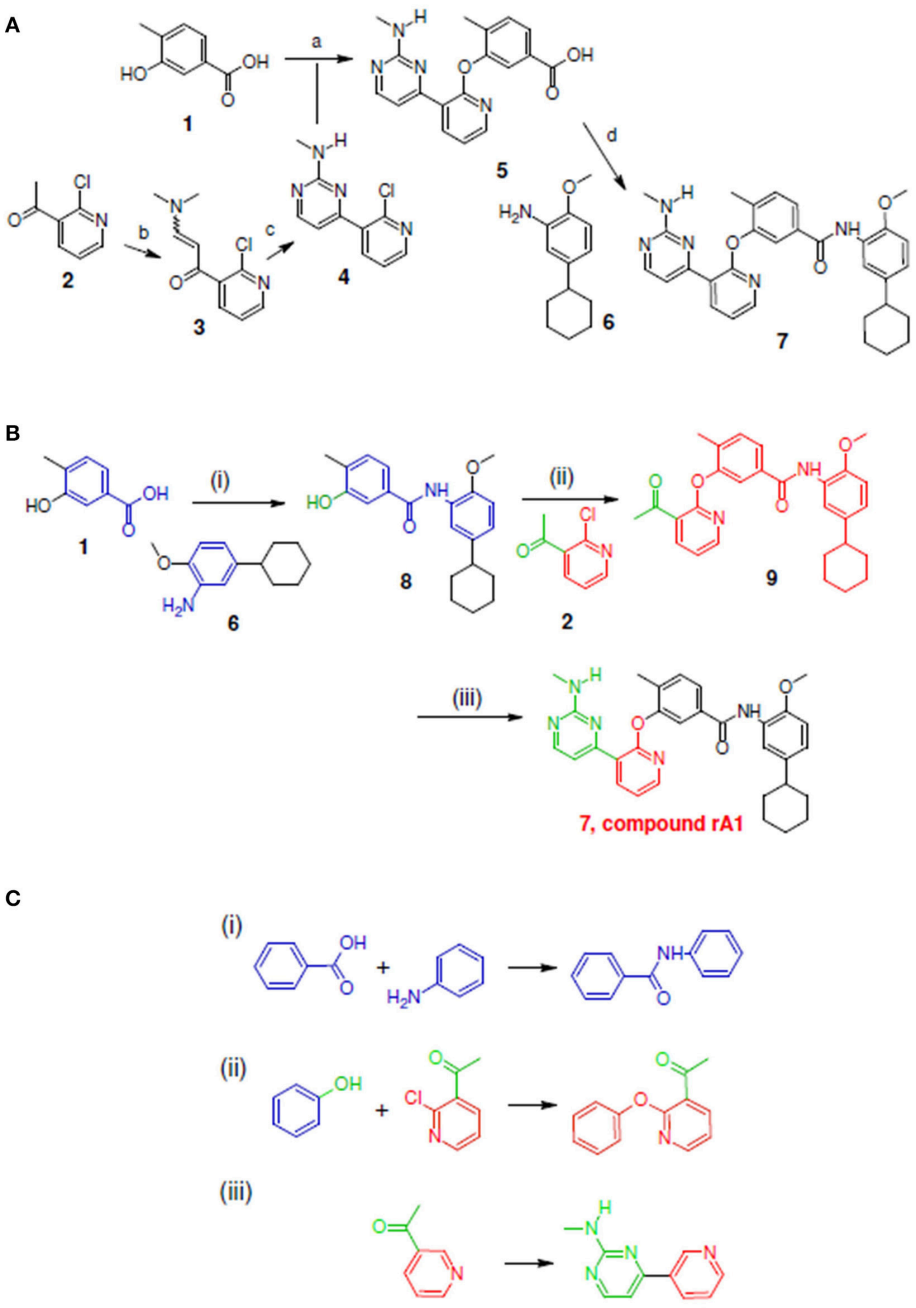
Synthetic routes for compound rA1 and sub-structure searching to identify atom arrangements (moieties) that are part of a chemical reaction rule within the LeadOp+R reaction database. **(A)** Synthetic routes with reagents and condition (a–d) from experimental studies (Hodous et al., 2007). **(B)** Synthetic routes and **(C)** matched reaction rules provided by LeadOp+R.

**Figure 6:**
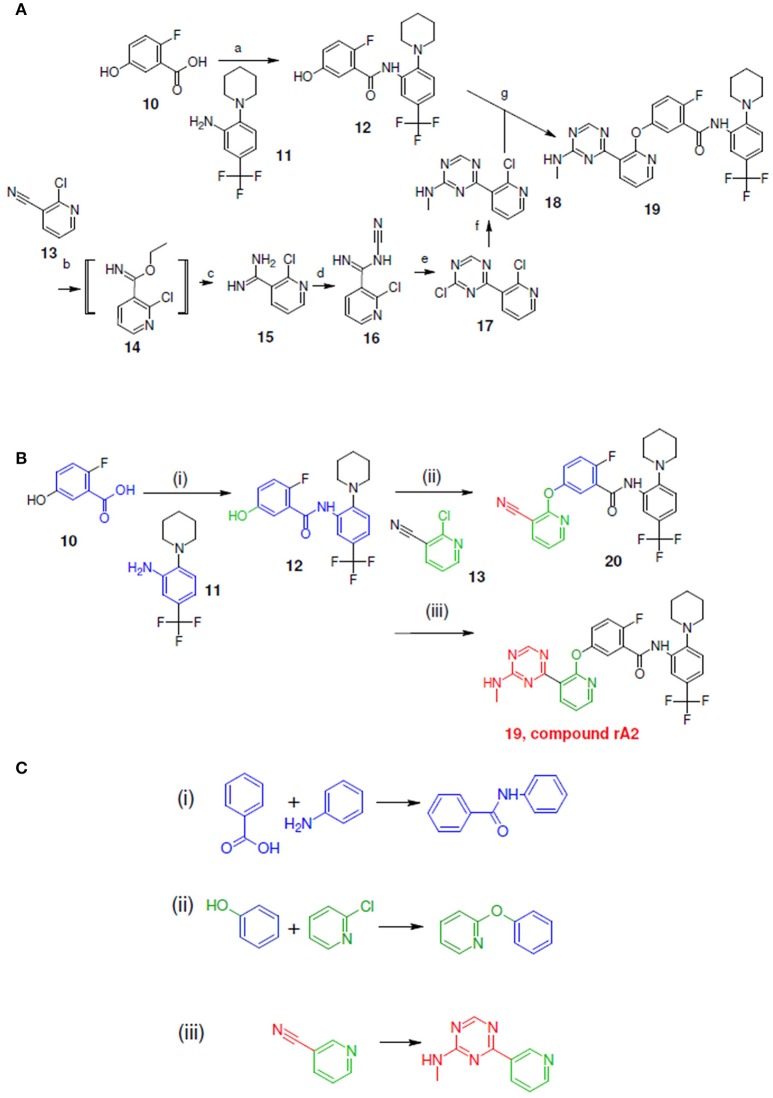
Synthetic routes for compound rA2 and sub-structure searching to identify atom arrangements (moieties) that are part of a chemical reaction rule within the LeadOp+R reaction database. **(A)** Synthetic routes with reagents and condition (a–g) from experimental studies (Hodous et al., 2007). **(B)** Synthetic routes and **(C)** matched reaction rules provided by LeadOp+R.

**Figure 7:**
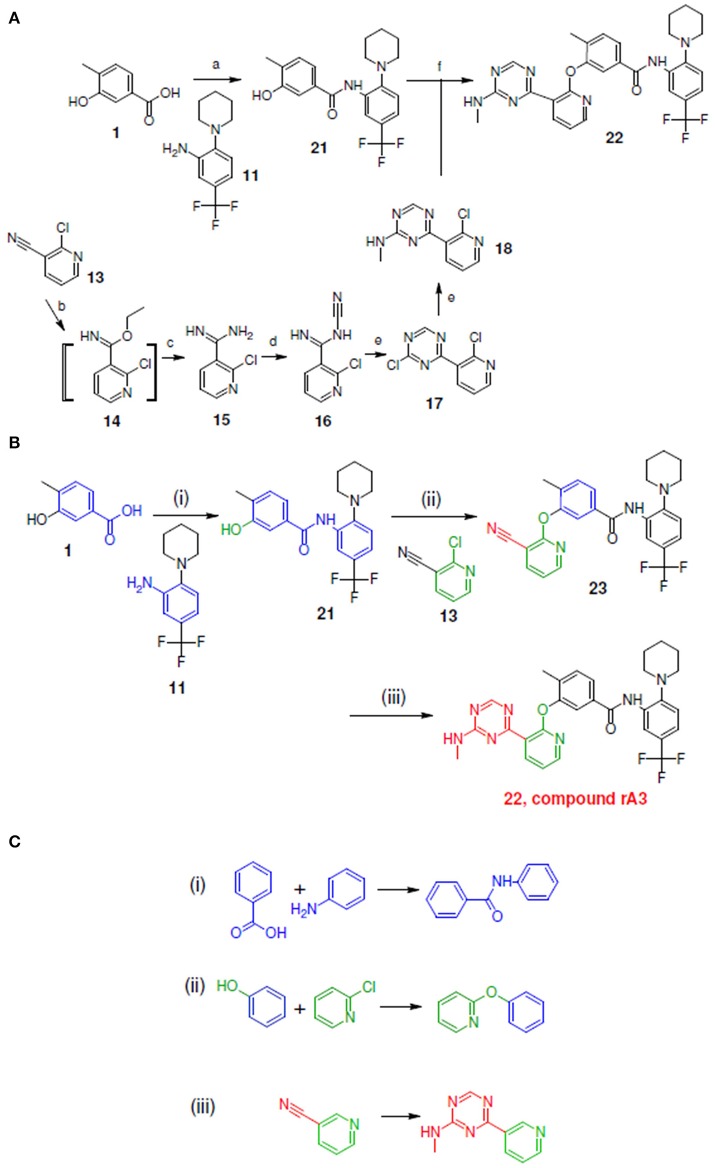
Synthetic routes for compound rA3 and sub-structure searching to identify atom arrangements (moieties) that are part of a chemical reaction rule within the LeadOp+R reaction database. **(A)** Synthetic routes with reagents and condition (a–f) from experimental studies (Hodous et al., 2007). **(B)** Synthetic routes and **(C)** matched reaction rules provided by LeadOp+R.

For Tie-2 kinase inhibitors, favorable interactions occur between the ligand and the specific receptor residues Glu 872, Asp 982, Phe983, Ala905, and Glu903 (see Figure 4A). In this example, these interactions are selected as preferred inhibitor-receptor interactions for LeadOp+R to optimize based on the provided query molecule in a selective and systematic process. Experimental synthetic routes from the literature (Hodous et al., 2007; Figures 5A, 6A, 7A) and the reaction routes suggested by LeadOp+R (Figures 5B, 6B, 7B) to generate compound rA1, rA2, and rA3 are summarized below to demonstrate how LeadOP+R suggests the synthetic reaction routes that are similar to those proposed by organic and medicinal chemists. Matched reaction rules are listed to the right of Figures 4C, 5C, 6C with details of each synthetic step identified by LeadOp+R, for each product, described below.

Figure 5A illustrates the experimental reactions required to synthesize compound rA1 (compound **7**) by reacting **5** (which was generated through transforming **2** into **4**) followed by reacting **1** with **6**. To compare LeadOp+R's suggested virtual synthesis of compound rA1 to proven synthetic routes; we compared the key reaction rules from experimental synthetic steps in the literature.

Figure 5B shows the LeadOp+R suggested synthetic routes to generate compound rA1 using the selected and preferred inhibitor-receptor interactions that allowed LeadOp+R to selectively and systematically optimize the query molecule. Initially, compound **1** was identified as the first reactant by searching all building blocks with the preserved fragment. LeadOp+R then proceed to produce product **8** by coupling **1** with **6** with the reaction rule (i) that conserves the preferred interaction with Glu872 specified. The reaction rule suggested by LeadOp+R matched the synthetic steps in the literature that forms compound **7** by combining compound **5** and fragment **6**. Next, product **8** was considered as the reactant to interact with compound **2** to generate product **9** by growing molecules with preferred interaction toward Phe983. The second reaction rule (ii) suggested by LeadOp+R lead to product **9** that matched the same synthetic steps as those in the literature to synthesize compound **5** by reacting **1** with **4**. It is interesting to note that at this step, the structure marked in red is the current structure **9**, the same partial structure highlighted in red within the final product **7** (compound rA1) in the experimental synthesis. LeadOp+R continued the recursive optimization toward the cavity near Phe983 and Ala905 to transform **9** to **7** (compound rA1) with the third reaction rule, Figure 5C. This reaction route suggested by LeadOp+R also matches the experimental synthetic route in the literature to transform **2** into **4**. To this end, LeadOp+R has successfully optimized the query compound rA to compound rA1 and suggested corresponding synthetic routes. In this example, we demonstrated how LeadOp+R controls the synthetic flow by extending the molecules with preferred interactions, available building blocks and associated reactions rules to reach fragment based optimization and synthetic accessible. Thus, the sequence of reactions to “grow” molecules may not be the same as those verified in experimental synthesis.

Figure 6A shows the experimental reaction to synthesize compound rA2 (compound **19**) by reacting **18** (which was generated through the transformation of **13**–**18**) with **12** (which was generated through the reaction of **10** with **11**). To compare the LeadOp+R suggested virtual synthesis route for compound rA2 with the experimental synthetic route, we compared the key reaction rules from the experimental synthetic steps in the literature with the LeadOp+R suggested synthetic routes.

Figure 6B shows the LeadOp+R suggested synthetic routes for compound rA2, using the selected and preferred inhibitor-receptor interactions to optimize the query molecule in a selective and systematic manner. Initially, a hydroxy benzoic acid of **10** was identified as the first reactant by searching all building blocks with the preserved fragment. LeadOp+R then proceed to suggest product **12** by reacting **10** with **11** via the first reaction rule (i) that preserves the ligand's interaction with Glu972 of the active site. The reaction rule suggested by LeadOp+R matched the synthetic steps in the literature that forms compound **12** from compounds **10** and **11**. Next, product **12** was considered as the reactant to react with compound **13** to generate product **20**, by growing molecules with preferred interaction toward Phe983. The second reaction rule (ii) generates product **20** and the reaction route suggested by LeadOp+R matches the synthetic steps in the literature to synthesize compound **19** through the reaction of **12** with **18**. LeadOp+R's recursive optimization continues toward the cavity near Phe983 and Ala905 to transform **20** to **19** (compound rA2) via the third reaction rule (iii), Figure 6C. This reaction route suggested by LeadOp+R also matched the experimental synthetic step in the literature to transform compound **13**–**18**.

Figure 7A shows the experimental reaction to synthesize compound rA3 (compound **22**) by reacting **21** (which was generated through the reaction of **1** with **11**) with **18** (which was synthesized from **13**). To compare LeadOp+R's suggested synthesis route for compound rA3 with the experimental synthetic routes, we compared the key reaction rules from the experimental synthetic steps in the literature with the LeadOp+R suggested synthetic routes.

Figure 7B depicts the LeadOp+R suggested synthetic routes to generate compound rA3, using the selected and preferred inhibitor-receptor interactions to optimize the query molecule. Initially, compound **1**, a hydroxybenzoic acid, was identified as the first reactant by searching all building blocks with the preserved fragment indicated in red, Figure 7B. LeadOp+R then proceeded to produce compound **21** by reacting **1** with **11** via the first reaction rule (i) directing the growth of the compound (inhibitor) toward the preferred ligand interaction with Glu972. The reaction rule suggested by LeadOp+R matched the synthetic steps in the literature that forms compound **21** via the transformation of compound **1** with fragment **11**. Next, product **21** was reacted with compound **13** to generate product **23**, growing the transformed molecule toward Phe983. The second reaction rule (ii) generated product **22** as suggested by LeadOp+R and matches the same synthetic steps as those in the literature to synthesize compound **22** through the reaction of compound **21** with fragment **18**. The recursive optimization of the initial query compound toward the cavity near Phe983 and Ala905 by LeadOp+R transformed compound **23** to **22** (compound rA3) with the third reaction rule (iii) as illustrated in Figure 7C. This reaction rule, suggested by LeadOp+R, also matches the experimental synthetic step in the literature to transform **13**–**18**.

LeadOp+R has successfully optimized the query compound rA to compounds rA1, rA2, and rA3 with synthetic routes that match experimental synthetic routes for each compound. Through the systematic synthesis and constant evaluation of intermediate products via group efficiency, LeadOp+R searched each product and discovered higher binding inhibitors. Increased hydrophobic interactions between compound rA1 and the receptor were observed between the compound's aromatic group that resides in the EHP pocket (Figure 4B) and the methylpyrimidine. This corresponds to the experimental results and rA1 exhibits stronger inhibitor potency than compounds rA2 and rA3.

In the example of Tie-2 inhibitor design, LeadOp+R demonstrates its ability to control the synthetic flow by extending the query molecules to optimize the preferred ligand-receptor interactions while using the available building blocks and associated reactions rules to find the most feasible synthetic accessibility.

### LeadOp+R for human 5-lipoxygenase inhibitor

#### Structure-based lead optimization with synthetic routes

**Figure 8:**
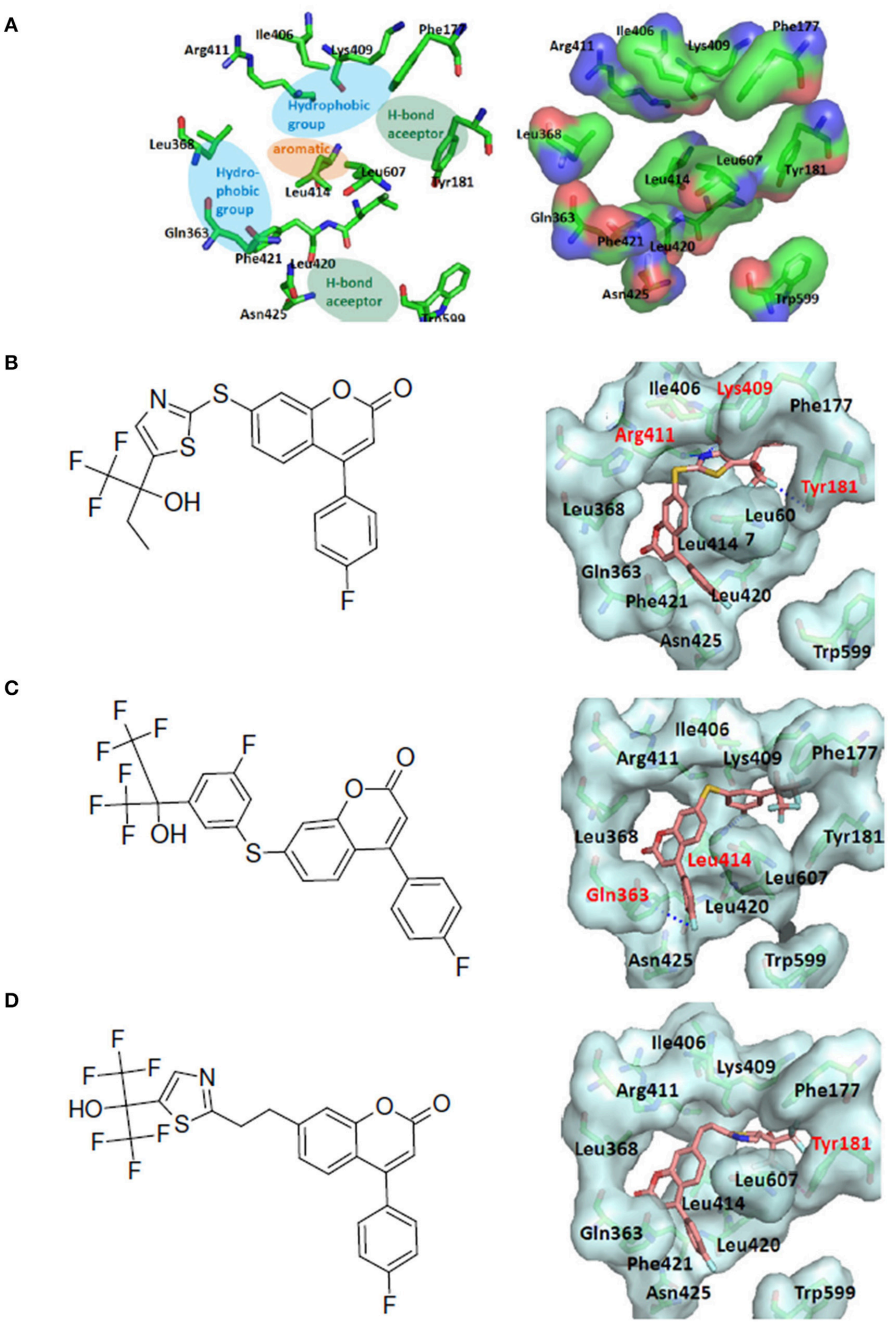
LeadOp+R result for the 5-LOX kinase model system. **(A)** Schematic representation of the human 5-LOX active site (left) and the binding pocket (right). The purported pharmacophores of the binding site of 5-LOX involving two hydrophobic groups (blue ovals), two hydrogen bond acceptors (green ovals), and an aromatic ring (orange oval) for ligand binding at the binding cavity. **(B–D)** Chemical structure (left) and MDS result (right) of the generated compound rB1 **(B)**, the generated compound rB2 **(C)**, and the generated compound rB3 **(D)**. Carbon atoms are colored pink. Amino acid residues that participate in hydrogen-bonding interactions (labeled red) with the proposed compound within the binding site are depicted with gray molecular surfaces.

The human 5-Lipoxygenase (5-LOX) enzyme with the well-known 5-LOX inhibitors was selected as the second LeadOp+R test case. To design better 5-LOX inhibitors, structural insight of the 5-LOX active site and its associated interactions with ligands would be helpful; therefore we selected a theoretical model (comparative/homology protein structure/model) of 5-LOX (Charlier et al., 2006) that has good agreement with mutagenesis studies (Hammarberg et al., 1995; Schwarz et al., 2001). The proposed active site of 5-LOX forms a deep and bent cleft (channel) that extends from Phe177 and Tyr181 at the top of the cleft to the Trp599 and Leu420 amino acid residues at the bottom of the cleft (shown in Figure 8A). Most of the residues lining the cleft are hydrophobic with several *key* polar residues (Gln363, Asn425, Gln557, Ser608, and Arg411) distributed along the channel with the ability to interact with the ligand during the binding process. A small side pocket off of the main channel is composed of hydrophobic residues (Phe421, Gln363, and Lue368) and it is postulated that the lipophilic interactions between the ligand and receptor may enhance activity. The purported major pharmacophore interactions needed for a ligand to bind to 5-LOX includes: (i) two hydrophobic groups, (ii) a hydrogen bond acceptor, (iii) an aromatic ring, and (iv) two secondary interactions. The two secondary interactions are between the ligand and an acidic moiety (amino acid residue) and a hydrogen bond acceptor within the binding pocket of the receptor. The hydrogen bond acceptor of the ligand most likely interacts with the key anchoring points of the receptor (Tyr181, Asn425, and Arg411) to form hydrogen bonds, while Leu414 and Phe421 form a hydrophobic interaction between the ligand and the binding cavity (Charlier et al., 2006).

The 5-LOX inhibitor, compound 7 in the literature (Ducharme et al., 2010), was selected as our initial query molecule (denoted as compound rB in this study), which had a biologically determined IC_50_ value of 145 nM. Compound rB was docked into the 5-LOX computationally derived binding site and the lowest energy conformation was submitted to LeadOp+R. This selected pose (conformation) possesses similar ligand-receptor interactions as previously reported (Charlier et al., 2006). The oxochromen ring favorably interacts with the hydrophobic residue Leu414 (CH-π interaction) in the middle of the cavity, while the fluoro phenyl group extends into the hydrogen-bond acceptor region in the lower cleft of the active site. The docked conformation of compound rB was selected as the reference inhibitor with the oxochromen ring serving as the template structure.

To evaluate our algorithm, we compared all of the LeadOp+R generated compounds for 5-LOX to the analogs described in the literature and found that six of the LeadOp+R proposed compounds have been synthesized and their biological activities measured (Schwarz et al., 2001). The inclusive synthesis of products at each step combined along with systematically examining the interactions of the proposed compounds with the receptor generated six compounds with more potent IC_50_ values than the original compound (compound rB). All the LeadOp+R generated compounds were energy minimized within the active site of 5-LOX and then ranked based on the predicted binding energy of the complex and the suggested priority has the same trend as the IC_50_ potency values from the experimental study (Schwarz et al., 2001). In this study of 5-LOX inhibitor design, three compounds (denoted as compounds rB1, rB2, and rB3) of the nine LeadOp+R generated compounds, were selected for further investigation. For these three compounds detailed synthetic information (Ducharme et al., 2010) and inhibition potency is available from the literature (Ducharme et al., 2010). Additionally, these three compound rB1, rB2, and rB3 have a higher potency than the query compound rB and their suggested priority, based on predicted binding energy, as well as a similar IC_50_ trend. Depicted representations of the compounds rB1, rB2, and rB3, the corresponding inhibition data from the biological experiments, and their predicted binding energy are listed in Table 2.

**Table 2:** Rank of the proposed LeadOp+R compounds based on the calculated binding energy, inhibition contraction (IC_50_) of 5-LOX from the literature (Ducharme et al., 2010).

**Rank**	**Structure**	**Inhibition IC_50_ (nM)**
Query	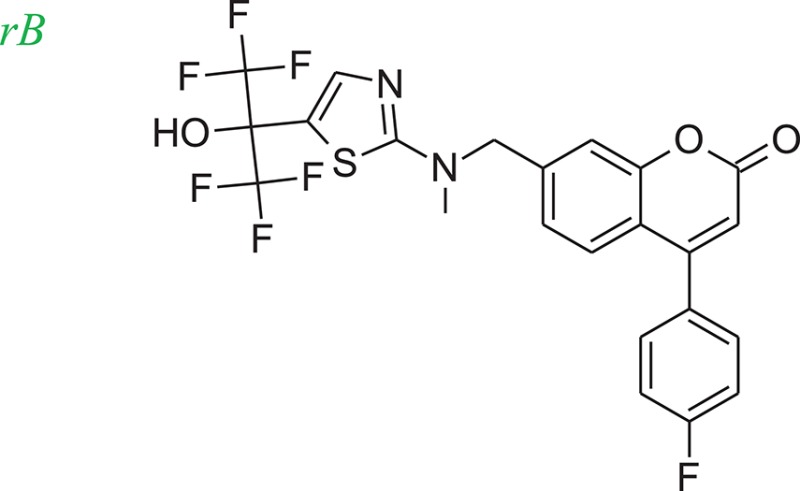	145
52	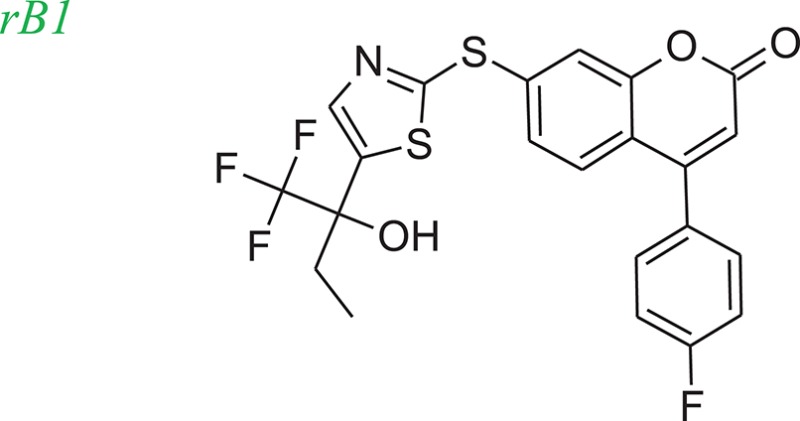	7 ± 2
107	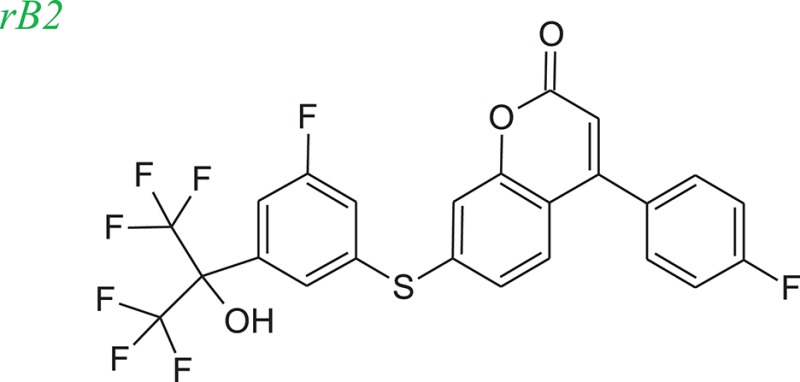	27 ± 16
297	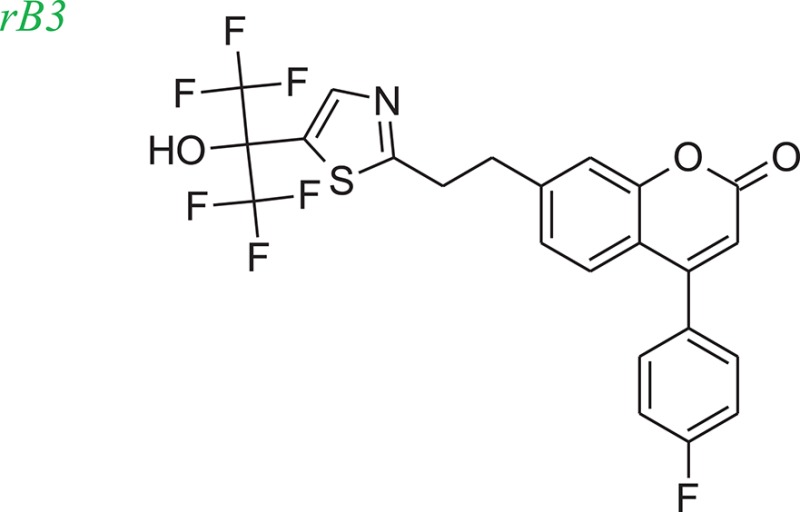	64 ± 3

*All proposed compounds have a higher IC_50_ value than the query compound and the suggested priority of the three new compounds (out of 419) have a similar trend as the IC_50_ potency values*.

Molecular dynamics simulation studies were performed with the final poses of compounds rB1, rB2, and rB3 with respect to 5-LOX. The unique low-energy conformations of the complexes, from the last 50 ps of the MDS (50 configurations), are shown in Figures 8B–D.

The interactions of compounds rB1, rB2, and rB3 all reside within the hydrophobic pocket and contain participate in hydrogen bonding interactions between the oxygen or nitrogen atoms of the thiazol group with Lys409 and Tyr181. For compounds rB1 and B3, the fluoro group extends to the hydrogen-bond acceptor in the upper domain of the active site and interacts with Lys409. In addition, the oxochromen ring is in close proximity to Leu414 and is potentially an important CH-π contact as indicated in the literature (Charlier et al., 2006). Also, the thiazole structure of compound rB1 interacts with the 5-LOX hydrophobic residues Leu420 and Leu607 and it has been suggested that these interactions improve ligand binding via complementary hydrophobic interaction between the ligand and receptor. Additionally, favorable interactions occur between the fluoro group and residues Lys409, Arg411, and Tyr181. These contributions to the ligand-protein binding probably accounts for compound rB1's better inhibition compared to compounds rB, rB2, and rB3. These optimized results indicate that hydrogen bonding and hydrophobic interactions are important for ligands binding to and inhibiting, 5-LOX as previous report (Hodous et al., 2007).

#### Synthetic routes suggested by LeadOp+R

**Figure 9:**
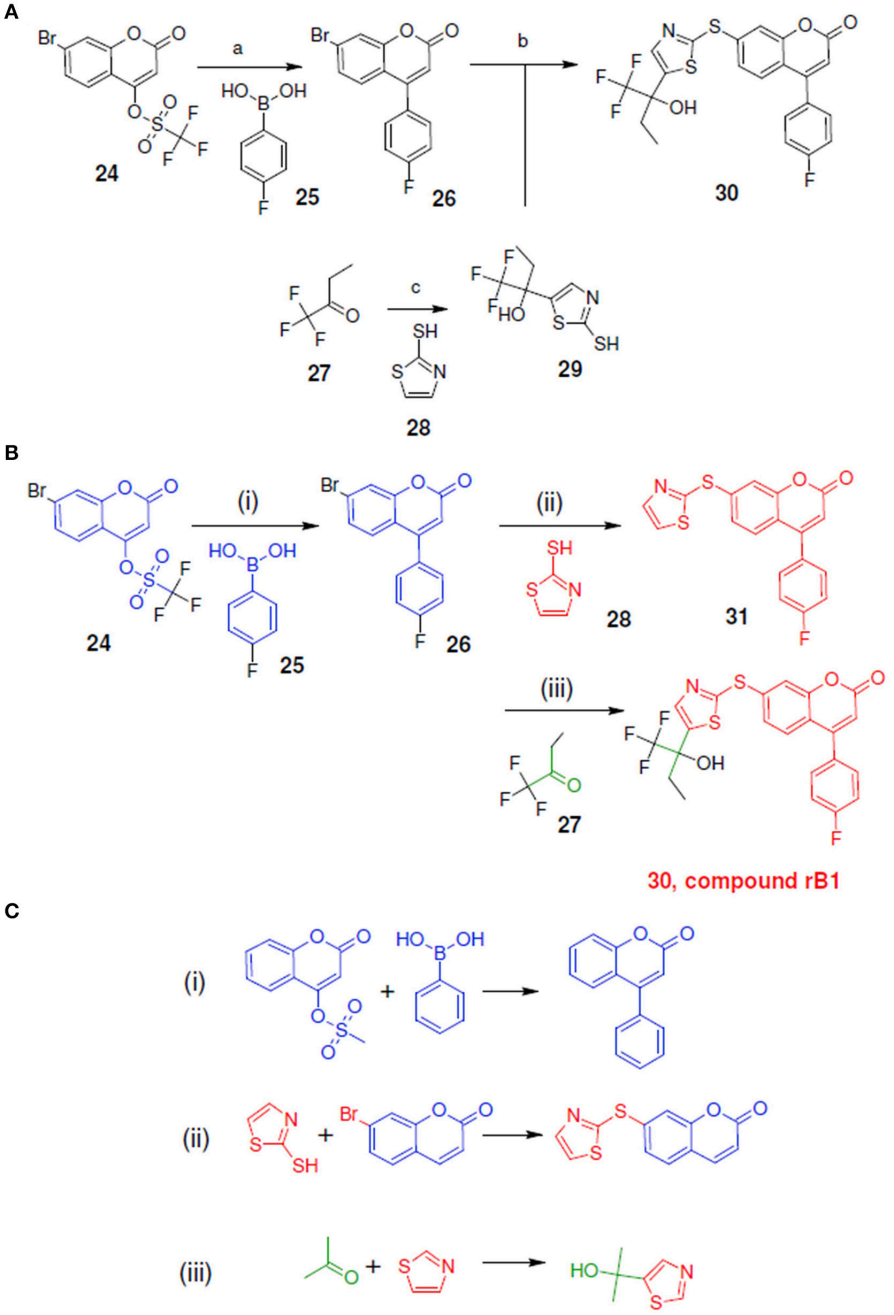
Synthetic routes for compound rB1 and sub-structure searching to identify atom arrangements (moieties) that are part of a chemical reaction rule within the LeadOp+R reaction database. **(A)** Synthetic routes with reagents and condition (a–c) from experimental studies (Ducharme et al., 2010). **(B)** Synthetic routes and **(C)** matched reaction rules provided by LeadOp+R.

**Figure 10:**
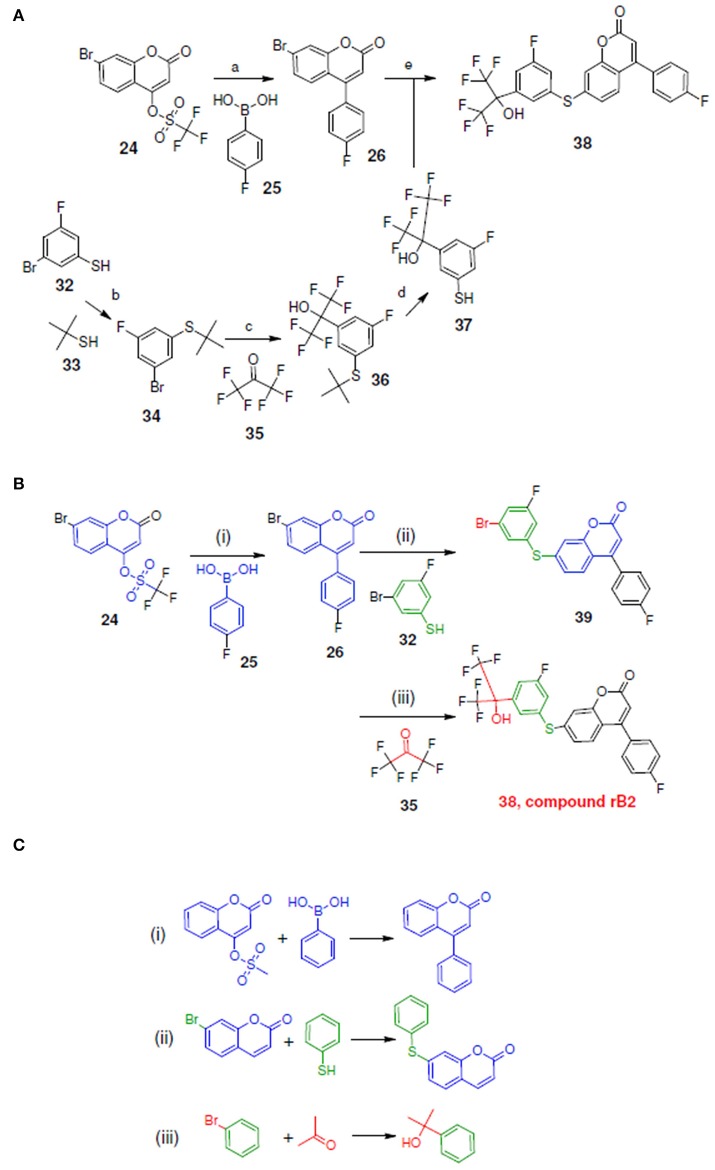
Synthetic routes for compound rB2 and sub-structure searching to identify atom arrangements (moieties) that are part of a chemical reaction rule within the LeadOp+R reaction database. **(A)** Synthetic routes with reagents and condition (a–e) from experimental studies (Ducharme et al., 2010). **(B)** Synthetic routes and **(C)** matched reaction rules provided by LeadOp+R.

**Figure 11:**
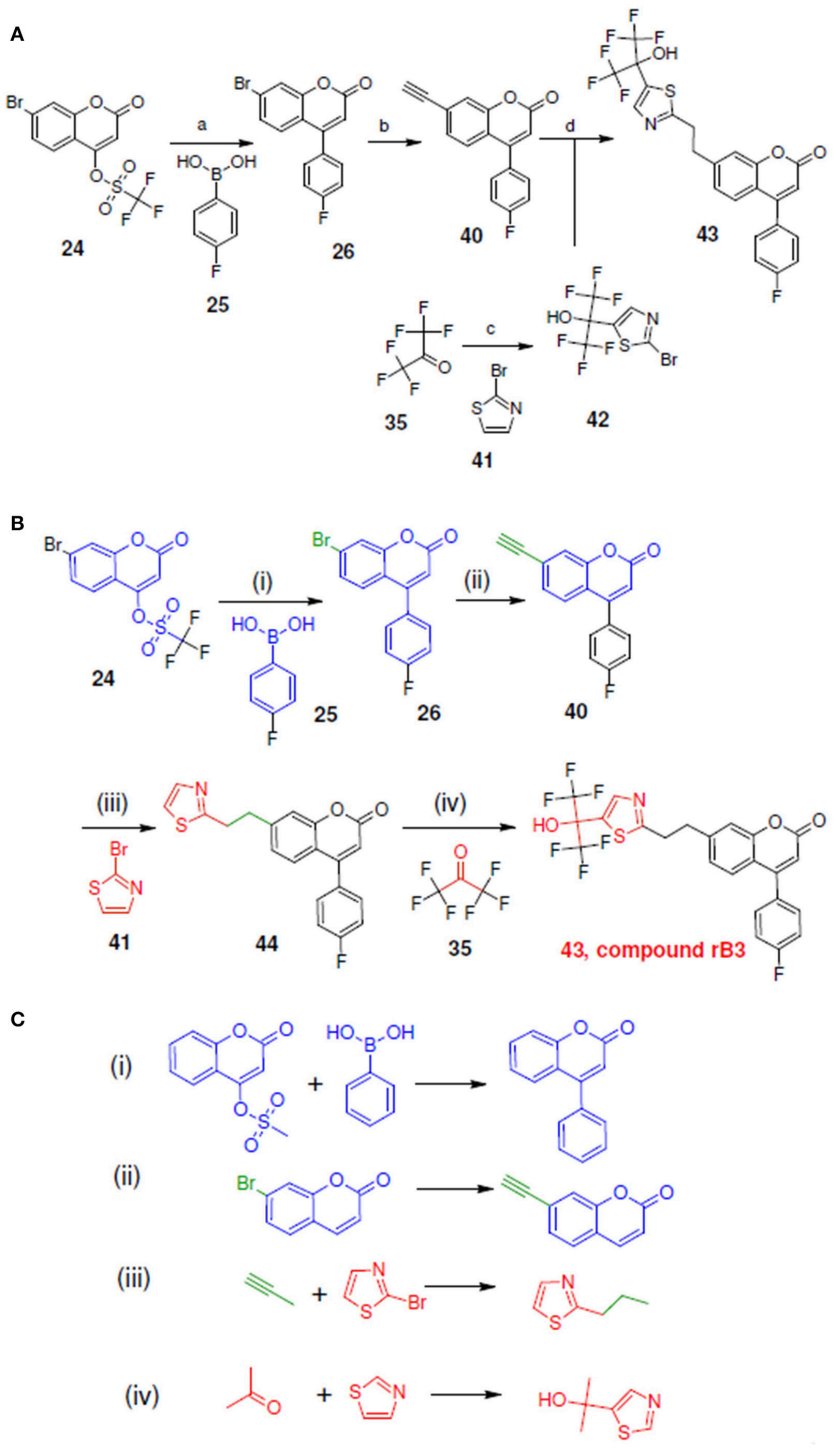
Synthetic routes for compound rB3 and sub-structure searching to identify atom arrangements (moieties) that are part of a chemical reaction rule within the LeadOp+R reaction database. **(A)** Synthetic routes with reagents and condition (a–d) from experimental studies (Ducharme et al., 2010). **(B)** Synthetic routes and **(C)** matched reaction rules provided by LeadOp+R.

The favorable interactions between inhibitors and 5-LOX, as stated in the literature, are two hydrogen-bond acceptor interactions within the binding pocket (including ligand interactions with Asn425 and Tyr181), two hydrophobic interaction pockets (including ligand interactions with Leu368, Gln363, Phe421, Arg411, Ile406, Lys409, and Phe177), and aromatic interactions (between the ligand and residues Leu414 and Leu607). In this example, ligand interactions with Asn425, Leu414, Leu607, and Tyr181 are indicated as “preferred” inhibitor-receptor interactions for LeadOp+R to selectively and systematically optimize. Experimental synthetic routes from the literature (Schwarz et al., 2001) (Figures 9A, 10A, 11A) and the synthetic reaction routes suggested by LeadOp+R (Figures 9B, 10B, 11B) to generate compound rB1, rB2, and rB3 are summarized below. To demonstrate LeadOp+R's ability to suggest reaction routes similar—or exactly the same—to those proposed and executed by synthetic chemists, the matched reaction rules are listed to the right of Figures 9C, 10C, 11C. Details of each synthetic step, identified by LeadOp+R for each product (proposed compounds/inhibitor), are described below.

Figure 9A shows the experimental reaction route (Schwarz et al., 2001) to synthesize compound rB1 (compound **30**) by reacting compound **26** (which was generated through the reaction of **24** with **25**) with **29** (which was generated through the reaction of **27** with **28**). To compare the LeadOp+R suggested synthesis with the experimental synthetic route for compound rB1, we compared the key reaction rules for the experimental synthetic steps in the literature with those suggested by LeadOp+R.

Figure 9B shows the LeadOp+R suggested synthetic routes to generate compound rB1 using the selected preferred inhibitor-receptor interactions. Compound **24** was identified as the initial reactant by searching all the available building blocks and preserving the molecular fragment. LeadOp+R suggested product **26** by reacting **24** with **25** with the first reaction rule (i) “growing” the compound toward the preferred interaction with Asn425. The reaction rule suggested by LeadOp+R matches the synthetic steps in the literature that yields compounds **26**, **24**, and **25**. Next, product **26** was considered as the reactant to interact with compound **28** to generate product compound **31** by extending the ligand toward preferred interactions with Leu414. The second reaction rule (ii) to generate compound **31**, as suggested by LeadOp+R, matches the synthetic routes presented in the literature to synthesize the thioether bond in compound **30** through the reaction of **26** with **29**. It should be indicated that in this step, the structure marked in red is compound **31** and it is the same as the partial structure denoted in red for the final product **30** (compound rB1) in the experimental synthesis. The recursive optimization continues via LeadOp+R toward the cavity near Ile406 and the synthesis of compound **30** (compound rB1) by reacting **31** with **27** and the third reaction rule (iii) in Figure 9C. The LeadOp+R suggested reaction route also matches the experimental synthetic step in the literature to synthesize compound **29** through the reaction of **27** with **28**. To this end, LeadOp+R has successfully optimized the query compound rB to compound rB1 and suggested feasible synthetic routes. In this example, we demonstrated LeadOp+R's control of the synthetic flow by extending the molecules to exploit preferred interactions, available building blocks, and associated reactions rules to achieve fragment based optimization and synthetic accessibility. For these reasons, the sequence of steps to “grow” molecules may not be the same as the published experimental synthesis.

Figure 10A depicts the experimental reaction scheme (Schwarz et al., 2001) to synthesize compound rB2 (compound **38**) by reacting **26** (which was generated through the reaction of **24** with **25**) with **37** (which was synthesized through a series of reaction starting with compound **32** to formed **37**). To compare LeadOp+R's suggested synthesis of compound rB2 to the experimental synthetic routes, we explored the key reaction rules of the experimental synthetic steps in the literature for the proposed compound.

Figure 10B shows the LeadOp+R selective and systematically suggested synthetic routes to generate compound rB2 based on the user specified preferred inhibitor-receptor interactions. Initially, compound **24** was identified as the first reactant by searching all building blocks with the preserved fragment. LeadOp+R then proceed to produce compound **26** by reacting **24** with **25** via the first reaction rule (i) that directs the suggested compound toward the preferred interaction with Leu414. The reaction rule suggested by LeadOp+R matches the synthetic steps in the literature for the synthesis of compound **26** from compound **24** and **25**. Next, product **26** was considered as the reactant to react with compound **32** to generate product **39**; again by growing the molecule toward the preferred interaction with Leu414. The second reaction rule (ii) to generate product **39** suggests the same synthetic steps as the literature to synthesize compound **38** by reacting **26** and **27**. The recursive optimization continues to explore the potential ligand interactions with Leu414 and Ile406 to generate compound **38** (compound rB2) by reacting **39** with **35** via the third reaction rule (iii) to synthesize compound **36** by the reaction of **34** and **35**, resulting in the final product compound rB2.

Figure 11A shows the experimental synthesis route (Schwarz et al., 2001) to synthesize compound rB3 (compound **43**) by reacting **40** with **42** (which was generated through the reaction of **35** with **41**). To compare the LeadOp+R suggested route to the experimental route for rB3, we look at the key reaction rules in the literature.

Figure 11B shows the LeadOp+R suggested synthetic routes for compound rB3 using the selected preferred inhibitor-receptor interactions. Initially, compound **24** was identified as the first reactant by searching all building blocks with the preserved fragment that is indicated in Figure 11B as the red structure. LeadOp+R proceeded to generate compound **26** by reacting **24** with **25** via the first reaction rule (i) suggested by LeadOp+R. Again, this methodology directs the growth of the new ligand toward the preferred interaction of the ligand interacting with Leu414. The synthetic reactions suggested by LeadOp+R match the synthetic steps presented in the literature that forms compound **26**. Next, product **26** was considered the reactant and transformed into product **40** by growing the ligand toward Ile406 of 5-LOX. The second reaction rule (ii) generates compound **40** and matches the synthetic steps discussed in the literature; compound **40** is identified as the same product that is discussed in the literature to synthesize compound **44**. Continuing the recursive optimization to initiate the ligand's interaction with Ile 406 and Tyr181 results in the third reaction rule (iii), Figure 11C, and leads to compound **43**. Compound **44** was identified as the reactant and reacted with **35** based on the fourth reaction rule (iv), generating compound **42** by reacting **35** with **41**.

LeadOp+R has successfully optimized the query compound rB into compounds rB1, rB2, and rB3 and has suggested corresponding synthetic route for each compound. Through systematic synthesis and evaluation of intermediates using group efficiency, LeadOp+R searches for “products” with higher calculated binding affinities and improved interactions with the receptor. The more hydrogen-bonding interactions between compound rB1's oxygen or nitrogen atoms of the thiazol group and the receptor (shown in Figure 8B) corresponds to the experimental results of stronger inhibitor potency then the proposed compounds rB2 and rB3. In the example of 5-LOX inhibitor design, we demonstrate LeadOp+R's ability to controls the synthetic flow by extending the ligands with preferred interactions, available building blocks, and associated reactions rules.

## Limitation

LeadOp+R is an optimization algorithm that starts with a query reactant (compound) and better lead optimization occurs when starting the optimization process with a good binder that is advantageously positioned in the binding site. The LeadOp+R algorithm does not consider experimental product yield rate, reaction rate, and reaction conditions of a chemical synthesis but does propose potential synthetic routes purely based on the chemical reaction rules contained in the chemical reaction database. However, incompatibility of the reaction with specific substituents in the core may happen, the proposed synthetic routes are meant to provide a fast, systematic, and preliminary suggestion based on general reaction—synthesis—rules and structure-based (receptor) ligand optimization. The diversity of the reactant database is a critical factor when searching for the participant reactants along with the number of different poses sampled at each reaction site. The greater the number and diversity of reaction rules and reactant available for LeadOp+R to explore—for the system of interest—the greater the possibility to identify and generate new compounds.

## Conclusion

In this work, we have implemented a structure-based lead optimization with synthetic accessibility algorithm called “LeadOp+R.” Two model systems, Tie-2 kinase and human 5-LOX enzyme with their associated inhibitors, were selected to demonstrate the abilities of the LeadOp+R algorithm. We demonstrated how a query molecule was enhanced through structured-based optimization and a potential synthetic route was proposed based on reaction rules extracted from in-house synthetic database. In the case of Tie-2, co-crystalized structure is available, while the human 5-LOX example was performed using a theoretical 5-LOX receptor model (comparative or homology protein model) and a known inhibitor. LeadOp+R generates a set of potential compounds that exhibit better-calculated inhibition, possess better efficiency score(s), along with providing synthesis routes based on published reaction mechanisms (contained in an in-house reaction and reactants database). The molecular dynamic simulation analysis further demonstrates that the generated structures preserve the important ligand-protein interactions as seen in the crystal structures or reported in the literature. For the proposed compounds with biological inhibition values (IC_50_) obtained from the literature, LeadOp+R calculated inhibition values corresponding (based on rankings) to the literature values. The interactions between the inhibitor and protein, as noted in the literature, were observed in the entire molecular dynamic simulation. Moreover, we identified fragments that created and retained ligand-receptor interactions that were stronger and more consistent than the original query compound; these fragments were selected based on reaction rules and discovered in our reactant database.

In short, LeadOp+R is an algorithm that can automatically optimize a query molecule based on reaction routes by searching and selecting reactants that can undergo chemical synthesis thus generating compounds with better binding affinity for the biological system (receptor) of interest. Additionally, users can indicate specific parts of the query compound to be optimized and assign the predicted binding space (portion of the binding site) for the generated products based on known ligand-receptor interactions or preference. LeadOp+R is an algorithm that cannot only optimize the lead compounds but also design favorable and practical synthetic routes based on known reaction mechanisms, leading to faster data feedback between experimental and computer-aided molecular design.

## Author contributions

YT: Initiated the concepts; F-YL and YT: Drafted, programmed, and performed the analysis on the projects; EE: Edited and gave comments on the work.

### Conflict of interest statement

EE was employed by exeResearch, LLC and The Chem21 Group, Inc. The other authors declare that the research was conducted in the absence of any commercial or financial relationships that could be construed as a potential conflict of interest.

## Footnotes

^1^“Sigma-Aldrich Chemie GmbH.” (Steinheim, Germany).

^2^“JChem.” 5.4.1.1 ed. (Budapest, Hungary: ChemAxon Ltd).

^3^“Marvin.” 5.4.0.1 ed., (Budapest, Hungary: ChemAxon Ltd).
